# Tonotopic and localized pathways from primary auditory cortex to the central nucleus of the inferior colliculus

**DOI:** 10.3389/fncir.2013.00077

**Published:** 2013-04-25

**Authors:** Craig D. Markovitz, Tien T. Tang, Hubert H. Lim

**Affiliations:** ^1^Department of Biomedical Engineering, University of MinnesotaMinneapolis, MN, USA; ^2^Department of Otolaryngology, University of MinnesotaMinneapolis, MN, USA; ^3^Institute for Translational Neuroscience, University of MinnesotaMinneapolis, MN, USA

**Keywords:** auditory cortex, inferior colliculus, plasticity, corticofugal, tonotopy, lemniscal

## Abstract

Descending projections from the cortex to subcortical structures are critical for auditory plasticity, including the ability for central neurons to adjust their frequency tuning to relevant and meaningful stimuli. We show that focal electrical stimulation of primary auditory cortex in guinea pigs produces excitatory responses in the central nucleus of the inferior colliculus (CNIC) with two tonotopic patterns: a narrow tuned pattern that is consistent with previous findings showing direct frequency-aligned projections; and a broad tuned pattern in which the auditory cortex can influence multiple frequency regions. Moreover, excitatory responses could be elicited in the caudomedial portion along the isofrequency laminae of the CNIC but not in the rostrolateral portion. This descending organization may underlie or contribute to the ability of the auditory cortex to induce changes in frequency tuning of subcortical neurons as shown extensively in previous studies.

## INTRODUCTION

Physiological studies have demonstrated the role of corticofugal projections for various forms of auditory plasticity. For instance, descending pathways can alter midbrain coding for sound localization (Nakamoto et al., 2008; Bajo et al., 2010) and frequency (Zhang et al., 2005; Suga, 2008). A large extent of research on corticofugal effects on auditory plasticity has focused on the interactions between primary auditory cortex (A1) and the central nucleus of the inferior colliculus (CNIC; Xiong et al., 2009), the main ascending and tonotopic region of the auditory midbrain. In particular, activation of A1 neurons most sensitive to a specific frequency can shift CNIC neurons to become more responsive to that frequency. This can be achieved through repetitive A1 stimulation combined with pure tone stimulation (Yan and Suga, 1998; Yan et al., 2005), by pairing A1 stimulation with activation of the nucleus basalis or other neuromodulatory pathways (Ma and Suga, 2003; Zhang et al., 2005), or using fear conditioning paradigms (Gao and Suga, 1998, 2000; Ji et al., 2001). Furthermore, inactivation of A1 has shown to prevent or limit frequency shifts in the CNIC (Ji et al., 2001; Zhang et al., 2005), further signifying the substantial role of the corticofugal system in inducing subcortical auditory plasticity.

The ability to induce fine frequency plasticity within the CNIC through activation of A1 descending pathways argues for the existence of a well-defined tonotopic corticollicular organization. However, based on anatomical studies, descending cortical projections from layer V (and layer VI to a lesser extent; Schofield, 2009; Bajo and King, 2013) of A1 terminate predominantly in non-lemniscal midbrain regions, including the dorsal (DNIC) and external (ENIC) nuclei of the inferior colliculus (IC), which correspond to poor or non-existent tonotopy (Aitkin et al., 1975; Faye-Lund, 1985; Huffman and Henson, 1990; Herbert et al., 1991; Winer et al., 1998; Winer, 2006; Malmierca and Ryugo, 2011). Traditionally, it was thought that there were no or minimal corticofugal projections to the CNIC. However, there has been increasing anatomical evidence that there are a reasonable number of projections from A1 to CNIC that are topographically organized (Andersen et al., 1980; Feliciano and Potashner, 1995; Saldana et al., 1996; Bajo and Moore, 2005; Coomes et al., 2005; Bajo et al., 2007; Xiong et al., 2009; Malmierca and Ryugo, 2011). One study using electrical stimulation of the CNIC and recording the antidromically activated neurons within A1 in guinea pig confirmed that the corticofugal projections to CNIC are precisely tonotopically organized in which A1 neurons only project to CNIC neurons within a similar frequency region (Lim and Anderson, 2007a). Considering that the corticocollicular projections are glutamatergic (Feliciano and Potashner, 1995), these findings across studies provide one way in which the corticofugal projections can potentially elicit excitatory and tonotopic effects within the CNIC and contribute to the fine frequency plasticity shown in previous studies. However, questions remain as to how this descending activation can cause neurons located in neighboring frequency regions of the CNIC to shift their tuning toward the frequency of the stimulated A1 neuron if the corticofugal projections are organized in a point-to-point tonotopic pattern. In addition, most of the corticocollicular neurons project to non-lemniscal midbrain regions with poor or non-existent tonotopy, which in turn can activate neurons across CNIC (Huffman and Henson, 1990; Jen et al., 2001). Thus, it is unknown from these previous studies if the descending neurons from A1 can actually elicit an excitatory and tonotopic activation pattern within the CNIC.

There have been several studies showing the effects of A1 electrical stimulation on neural firing in the IC in bats (Sun et al., 1989; Yan and Suga, 1996; Zhang and Suga, 1997, 2000; Jen et al., 1998, 2001) and, to a lesser extent, in cats (Massopust and Ordy, 1962; Mitani et al., 1983), rats (Syka and Popelar, 1984), mice (Yan and Ehret, 2001, 2002; Yan et al., 2005), and guinea pigs (Torterolo et al., 1998). These studies have demonstrated that cortical activation can result in excitatory and/or inhibitory effects within the IC. However, these studies either looked at residual effects (i.e., changes in tuning or responses to acoustic stimuli after electrical stimulation had ceased) or were not designed to systematically investigate the cortically induced activation patterns along the tonotopic and isofrequency dimensions of the CNIC. Based on one previous study in guinea pigs (Bledsoe et al., 2003), there appears to exist differences in excitatory and inhibitory patterns across the CNIC, but it not yet clear how these differences vary along and across the frequency laminae. Therefore, in this study, we investigated if electrical stimulation of A1 could induce responses systematically across the tonotopic axis of the CNIC, exciting not only neurons sensitive to the same frequency but also those in neighboring frequency regions that could enable subcortical shifts in frequency tuning. We also investigated if there was any spatial organization of A1 descending pathways along the isofrequency laminae of the CNIC by creating three-dimensional histological reconstructions of the midbrain.

## MATERIALS AND METHODS

### ANIMAL SURGERIES AND ELECTRODE IMPLANTATION

Experiments were performed on 20 young Hartley guinea pigs (295–410 g; Elm Hill Breeding Labs, Chelmsford, MA, USA) in accordance with policies of the University of Minnesota Institutional Animal Care and Use Committee. Each animal was anesthetized with an intramuscular mixture of ketamine (40 mg/kg) and xylazine (10 mg/kg) with 0.1 mL supplements every 45–60 min to maintain an areflexive state. Atropine sulfate (0.05 mg/kg) was administered periodically to reduce mucous secretions in the airway. Heart rate and blood oxygenation were continuously monitored via a pulse oximeter and body temperature was maintained at 38.0 ± 0.5°C using a heating blanket and rectal thermometer.

**FIGURE 1:**
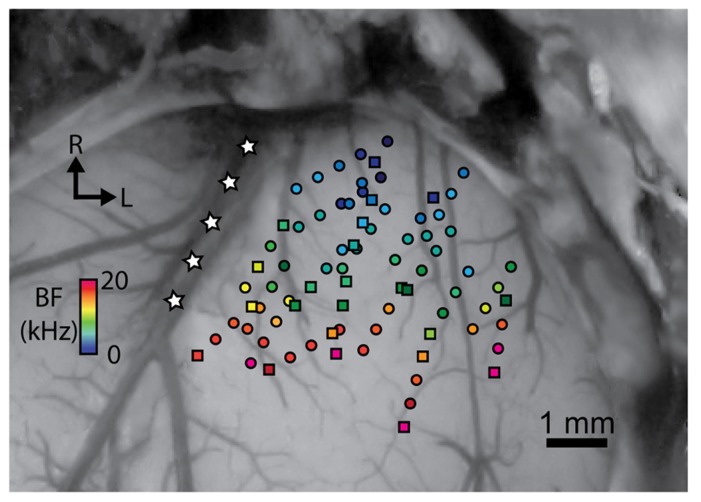
**Stimulation of sites at different locations spanning the isofrequency dimension of A1 results in excitation in the CNIC**. A dorsal view of the right A1 is shown that is approximately perpendicular to the cortical surface. A1 locations were normalized based on their relative distances from the pseudosylvian sulcus (labeled with white stars), bregma, and the lateral suture line. Site placements are color coded based on their best frequency (BF). Circles correspond to locations in which electrical stimulation resulted in excitation in at least one recording location in the CNIC, while squares are those that did not cause excitation on any of the CNIC recording sites for a given experiment. R, rostral; L, lateral.

After the animals were fixed in a stereotaxic frame (David Kopf Instruments, Tujunga, CA, USA) and a craniotomy was performed to expose the right auditory and visual cortices, two 32-site electrode arrays (NeuroNexus Technologies, Ann Arbor, MI, USA) were inserted via hydraulic micro-manipulators into the right A1 and CNIC. The A1 array consists of four 5 mm long shanks separated by 500 μm with eight iridium sites linearly spaced 200 μm (center-to-center) along each shank. Before each experiment, A1 electrodes sites were activated from iridium to iridium oxide via cyclic voltammetry for recording and stimulation capabilities (Lim and Anderson, 2007a), lowering the site impedances to approximately 0.1–0.3 MΩ. The array was placed perpendicular to the cortical surface and inserted to a depth of approximately 1.6 mm. The four shanks were arranged approximately along the tonotopic gradient of A1 (Redies et al., 1989; Wallace et al., 2000), which is shown in **Figure 1**. The CNIC array consists of two 10 mm long shanks separated by 500 μm with 16 iridium sites linearly spaced 100 μm along each shank. The array was inserted 45° off the sagittal plane through the occipital cortex into the CNIC to align it along the tonotopic gradient of the CNIC (Snyder et al., 2004; Lim and Anderson, 2006). CNIC site impedances ranged between 0.8 and 3.0 MΩ. After placement of the probes, the brain was covered with agarose to reduce swelling, pulsations, and drying during the recording sessions.

### RECORDING AND STIMULATION

Experiments were performed within a sound attenuating, electrically shielded room using custom software and TDT hardware (Tucker-Davis Technology, Alachua, FL, USA). All acoustic stimulation was presented to the animal’s left ear canal via a speaker coupled to a custom-made hollow ear bar. The speaker-ear bar system was calibrated using a 0.25” condenser microphone (ACO Pacific, Belmont, CA, USA).

Multi-unit neural data was recorded and sampled at a rate of 25 kHz, passed through analog DC-blocking and anti-aliasing filters up to 7.5 kHz, and digitally filtered between 0.3 and 3.0 kHz for analysis of neural spikes. Spikes were determined as voltages exceeding 3.5 times the standard deviation of the noise floor.

Electrical stimulation of A1 consisted of single biphasic, charge-balanced pulses (205 μs/phase, cathodic-leading) ranging from 4 to 32 μA in 2 dB steps at a rate of 2/s. All 32 A1 sites were stimulated at each level in a randomized pattern for 20 trials for each stimulus condition. Poststimulus time histograms (PSTHs) of the responses recorded at 32 CNIC sites following A1 stimulation were plotted for further analysis. When excitation was found in the CNIC in response to A1 stimulation, all analyses were performed using the lowest threshold cortical site along a given cortical shank, which was generally located at a depth of approximately 900–1500 μm and corresponds to layer V in the guinea pig cortex (Wallace et al., 2000; Lim and Anderson, 2007a). Typically, one array placement (i.e., four shank placements) was made in A1 and multiple array placements were made throughout the CNIC during each experiment. Each CNIC array placement (i.e., two shank placements) resulted in sites along each shank that were aligned along the tonotopic gradient of the CNIC. The CNIC array was then moved to multiple locations across the laminae during each experiment. The recording ground wire was positioned in the neck muscles and the stimulation ground needle was implanted into the brain tissue near the intersection of the midline and bregma.

### HISTOLOGY AND ELECTRODE SITE RECONSTRUCTIONS

A full explanation of the computer reconstructions of the midbrain for identifying the locations of CNIC sites was presented in a previous publication (Markovitz et al., 2012) and is only briefly described here. The CNIC array was dipped in a red fluorescent dye (3 mg Di-I per 100 μL acetone; Sigma-Aldrich, St. Louis, MO, USA) prior to its insertion into the brain. Immediately following each experiment, the animal was euthanized with an overdose (0.22 mL/kg) of Beuthanasia-D Special (active ingredients: pentobarbital sodium (390 mg/mL) and phenytoin sodium (50 mg/mL); Merck, Summit, NJ, USA) into the heart and decapitated. The brain was immersed in 3.7% paraformaldehyde for approximately 10 days. The midbrain was then blocked, cryosectioned into 60 μm thick sagittal slices, and fully reconstructed along with the electrode shank tracks (marked with the red Di-I stain) using computer software (Rhinoceros, Seattle, WA, USA). To create computer simulations of isofrequency laminae, the midbrains were three-dimensionally normalized to each other based on the size and orientation of the IC surface across animals, and the electrode tracks were superimposed within one standard midbrain. Three planes were identified perpendicular to the shank tracks and approximately correspond to low (2.0–3.2 kHz), middle (5.0–8.0 kHz), and high (10.0–16.0 kHz) frequency laminae. These laminae were chosen to give us a representative view of the isofrequency axis of the CNIC and were made to approximately correspond to two critical bands in thickness (Schreiner and Langner, 1997; Egorova et al., 2006; Malmierca et al., 2008). All neurophysiological data corresponding to a given frequency range was superimposed onto a “pooled” lamina, and the distance in the caudal–rostral and medial–lateral directions were normalized based on the most proximal site location in each direction. Though the actual laminae are curved and occupy an orientation that is somewhere between the medial–lateral and dorsal–ventral axes, we will use the “medial–lateral” notation for this dimension since this is what is commonly used in other physiological studies that have mapped properties across the isofrequency laminae of the CNIC (Schreiner and Langner, 1988; Ehret, 1997; Langner et al., 2002; Hage and Ehret, 2003).

Site locations in A1 were identified by imaging the exposed cortical surface with the inserted array shanks using a microscope-mounted camera (OPMI 1 FR pro, Zeiss, Dublin, CA). The shank locations across animals were then normalized based on their relative distances from the pseudosylvian sulcus, bregma, and the lateral suture line, as successfully performed in previous studies (Schreiner et al., 2000; Wallace et al., 2000; Eggermont and Roberts, 2004).

### DATA ANALYSIS

#### Acoustic-driven responses

Acoustic stimuli were presented to the animal’s left ear canal and acoustic-driven responses were recorded in A1 and the CNIC to determine the functional location of each electrode site. Pure tones (50 ms duration, 5 ms ramp/decay) of varying frequencies (0.6–38 kHz, 8 steps/octave) and levels (0–70 dB in 10 dB steps) were randomly presented (4 trials/parameter). The acoustic-driven spike rates were calculated for responses recorded in the CNIC (taken 5–60 ms after tone onset) and A1 (5–20 ms after tone onset) to create frequency response maps (FRMs) for each site. Best frequencies (BFs) were calculated from the FRMs as the frequency centroid at 10 dB above the visually determined threshold.

To verify the functional placement of our A1 array, FRMs with approximately equal BFs for each site along a single cortical shank confirmed that the array was inserted perpendicular to the cortical surface along a cortical column. Across shanks, increasing BFs along the rostrolateral to caudomedial direction and short response latencies of approximately 15 ms verified that our array was within A1, as shown in **Figure 1** (Wallace et al., 2000; Lim and Anderson, 2007b). High frequency (>20 kHz) A1 locations were generally avoided to prevent confusion with the shared high frequency border between A1 and the dorsocaudal cortical area (Wallace et al., 2000). To ensure that we positioned sites fully spanning the isofrequency dimension of A1, we initially mapped the cortical surface at the medial and lateral edges of A1 by recording and assessing FRMs and acoustic-driven properties that distinguish A1 from the non-A1 regions as described in previous studies (Redies et al., 1989; Wallace et al., 2000; Grimsley, 2008). The pseudosylvian sulcus (white stars in **Figure 1**) generally corresponds to the medial edge along the isofrequency dimension of A1. The lateral edge along the isofrequency dimension of A1 was identified by observing neural responses that were poorly tuned to pure tones or had long acoustic-driven latencies for locations beyond that edge. Array placements within the CNIC were confirmed by observing FRMs that systematically increased in BF with increasing depth (Lim and Anderson, 2007b; Markovitz et al., 2012). FRMs for sites outside of the CNIC in external regions of the IC typically exhibited broad and weak tuning and/or multiple FRM peaks and were excluded for the analysis in this paper.

#### Electrical stimulation threshold

The threshold level for CNIC activation in response to A1 stimulation was determined using signal detection theory (Green and Swets, 1966; Lim and Anderson, 2007b). Spike rate distributions for a given CNIC site in response to 20 trials of A1 stimulation were plotted for the “signal” condition (using a 30 ms window starting 4 ms after the electrical artifact) and the “noise” condition (using a 30 ms window before the electrical artifact) on the same axes. The signal time window was selected based on visual identification of the stimulus-driven activity across all PSTH responses. By adjusting a criterion spike rate level across the signal and noise distributions, the percentage of signal trials exceeding that criterion (correct hits) and that of noise trials (false alarms) were calculated and plotted for varying criterion levels to obtain a receiver operating characteristic (ROC) curve. The area under the ROC curve corresponds to the performance level for an ideal observer detecting a stimulus based on the signal and noise distributions in a two-alternative, forced-choice task. Using the area under the ROC curve for each stimulus level, a neurometric curve was plotted with performance levels ranging from 0.5 (chance) to 1.0 (perfect detection). Activation threshold was defined as the lowest current level that achieves at least a 76% performance level. This performance value was chosen because it sits on the steepest portion of the neurometric curve, making it a robust measure.

#### First-spike latencies

First-spike latencies for CNIC sites in response to A1 stimulation were calculated from the PSTHs by taking the first time bin to exceed 3.5 standard deviations above the pre-stimulus noise floor, and were visually confirmed to avoid any spurious fluctuations in the PSTHs. All CNIC latencies were determined at a suprathreshold current level of 2 dB above threshold.

For cases with more than one activated site along a CNIC shank in response to stimulation of an A1 site, two groups were used for latency comparison: (1) *BF-aligned*, consisting of the CNIC site with the closest BF to the stimulated A1 site, and (2) *BF-unaligned*, consisting of all other CNIC sites along the same shank showing a response. We then directly compared latencies between these two groups after a normalization procedure. We stimulated one site on a given A1 shank and recorded the responses on the sites across a CNIC shank, which we define as an *A1–CNIC shank pair*. Normalization was performed for each A1–CNIC shank pair in which the shortest latency across all sites along the CNIC shank was labeled as time 0 while the remaining latency values along that same shank were normalized relative to that time. This normalization procedure enabled us to combine latency values across different placements and animals and directly compare those values between the BF-aligned and the BF-unaligned groups. All statistical comparisons between different latency groups were performed using an unequal variance two-tailed *t*-test on ranked data with significance defined as *p* < 0.01 (Ruxton, 2006).

## RESULTS

### CNIC EXCITATION IS INDUCED VIA STIMULATION THROUGHOUT A1

Multi-unit neural activity across the tonotopic axis and along isofrequency laminae of the CNIC was measured in response to focal electrical stimulation (single pulses, 4–32 μA, 205 μs/phase) of deeper output layers of A1 using 32-site electrode arrays. For each experiment, the A1 array (4 shanks, 8 sites/shank) was inserted into one position, while the CNIC array (2 shanks, 16 sites/shank) was inserted into several positions with the shanks aligned along the tonotopic axis of the CNIC, providing an average of 4–5 sites along a given lamina per animal. A total of 2,746 CNIC sites were sampled with BFs ranging from 1.0 to 24.8 kHz. Focal electrical stimulation of 57 out of 80 locations fully spanning across the isofrequency dimension of A1 elicited activation on at least one site along a CNIC lamina (**Figure 1**). These data demonstrate that CNIC excitation can be induced via stimulation throughout most of A1.

### CORTICOCOLLICULAR PATHWAYS ARE TONOTOPIC

**FIGURE 2:**
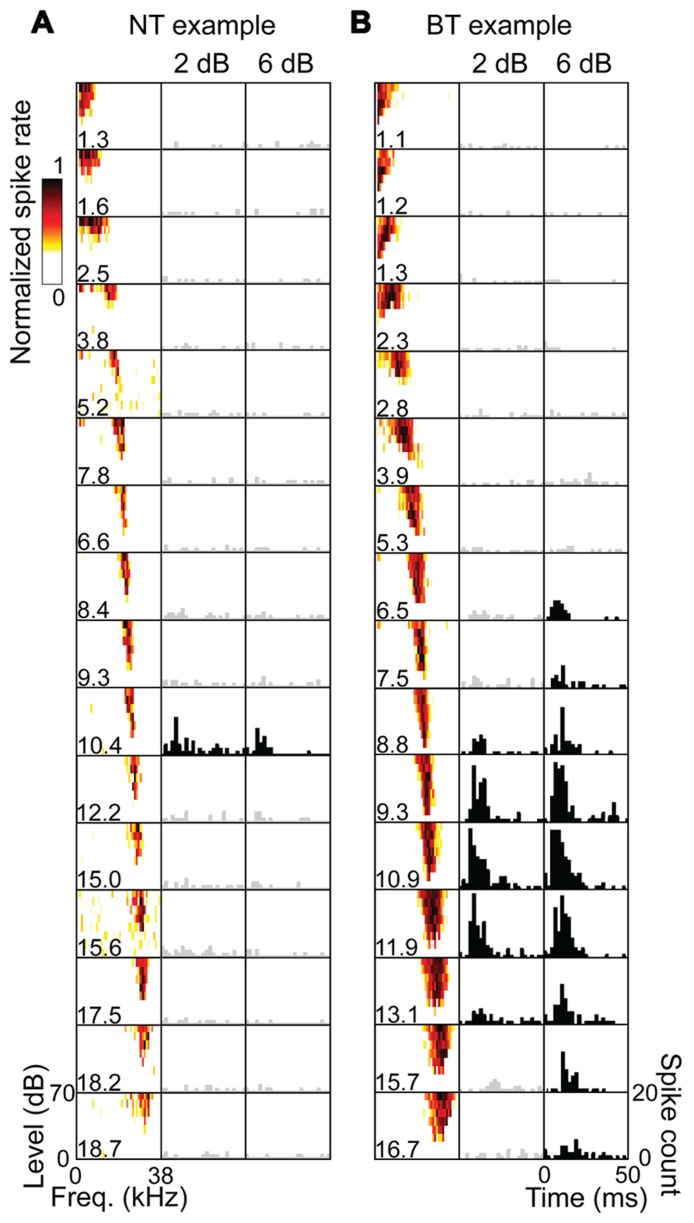
**Examples of NT and BT excitatory response patterns**. Each column represents the 16 sites of an electrode shank placed along the tonotopic gradient of the CNIC. Frequency response maps are labeled with the site’s best frequency (BF in kHz) in response to pure tone acoustic stimulation, showing a systematic increase in BF with depth. Poststimulus time histograms (PSTHs) are plotted for these same sites in response to stimulation of an A1 site (BF = 10.1 kHz) at 2 dB and 6 dB above electrical stimulation threshold. The PSTHs are summed across 20 trials with 0 ms corresponding to the onset of A1 stimulation. PSTHs in black show sites with activity that is significantly higher than spontaneous activity using a signal detection theory paradigm (see Materials and Methods). The NT **(A)** and BT **(B)** examples represent two different isofrequency placements of a CNIC shank for the same animal and A1 stimulation site. For the NT pattern, only a single CNIC site was activated in response to cortical stimulation as the stimulation level was increased to our maximum level of 32 μA. For the BT pattern, excitatory activity spread across a greater number of sites as the stimulation level was increased.

**FIGURE 3:**
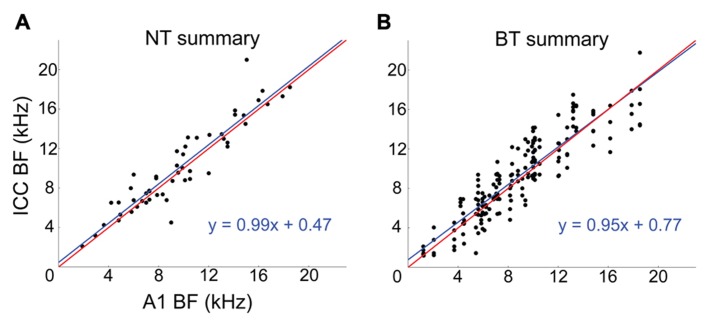
**Corticollicular pathways are tonotopically arranged**. Summary of tonotopic organization of NT **(A)** and BT **(B)** response patterns across experiments. The NT and BT data correspond to A1 stimulation at a level of 2 dB above threshold. Red lines represent a perfect linear correlation (i.e., exact tonotopic match) and blue lines are the linear best fit to the data. For the NT data, one A1–CNIC shank pair (i.e., one A1 location along the abscissa) corresponds to only one point along the ordinate. For the BT data, one A1–CNIC shank pair corresponds to several CNIC points along the ordinate.

Across the 20 experiments, we recorded from 87 CNIC shank positions which, combined with the 80 A1 stimulation locations (i.e., shank locations), resulted in a total of 346 A1–CNIC shank pairs. Of these, we found 88 A1–CNIC shank pairs that exhibited an excitatory activation pattern. When excitation was observed in the CNIC in response to stimulation of an A1 site, there were two highly distinct response patterns that emerged. We observed a narrow tuning (NT) type, in which typically only a single recording site out of 16 along a CNIC shank responded at all levels from threshold up to our maximum current level (**Figure 2A**). We also observed a broad tuning (BT) type, in which activation of multiple CNIC sites occurred at threshold with neural activity spreading across an increasing number of recording sites in the CNIC as we increased the stimulation level (**Figure 2B**). A summary of the NT and BT activation patterns across positions and animals is shown in **Figure 3**. In **Figure 3A**, only one point along the ordinate (i.e., CNIC site) is plotted for a given location along the abscissa (i.e., A1 site) since the NT pattern did not exhibit activation across more than one site along a CNIC shank. This NT pattern was tonotopic in which the stimulated A1 sites and the activated CNIC sites had similar BFs. The BT pattern was also tonotopically organized. However, the BT pattern consisted of activation across multiple sites along a CNIC shank in which several points along the ordinate are plotted for a given location along the abscissa as shown in **Figure 3B**. The data in **Figure 3** were plotted for a stimulation level of 2 dB above threshold. At this level, the BT pattern typically exhibited activity across 3–6 CNIC sites (frequency span – mean: 0.71, SD: 0.48 octaves).

### NEIGHBORING CNIC FREQUENCY REGIONS ARE ACTIVATED BY A1 STIMULATION

For the BT response pattern, A1 stimulation activated several CNIC sites that had BFs different from the BF of the stimulated A1 sites. In a previous study in guinea pig that stimulated the CNIC and recorded the antidromically activated spikes within A1 (Lim and Anderson, 2007a), it was shown that A1 neurons only project to CNIC neurons with a similar BF. This monosynaptic projection from A1 to CNIC could explain the BF-aligned activation observed for both the NT and BT patterns. However, it cannot explain the BF-unaligned sites activated for the BT pattern.

To gain further insight into these different activation patterns, we analyzed the first-spike latencies of CNIC responses to A1 stimulation. Comparing the first-spike latencies for the BF-aligned NT pattern (mean: 8.1, SD: 2.0, range: 5–12 ms) with only the BF-aligned sites for the BT pattern (mean: 7.2, SD: 1.5, range: 4–10 ms) resulted in no statistical difference (*p* = 0.091). These latencies were consistent with the published antidromic latencies of 2–10 ms (Lim and Anderson, 2007a) when accounting for the additional synaptic delay within the CNIC to record the postsynaptic spikes elicited by A1 stimulation. Thus, the BF-aligned activation for both the NT and BT patterns could be elicited through the monosynaptic projections from A1 to CNIC. We next compared the first-spike latencies between the BF-aligned and BF-unaligned sites for the BT pattern. The latencies, normalized to the fastest projection for each A1–CNIC shank pair (see Materials and Methods), were significantly shorter (*p* = 0.003) for BF-aligned (mean: 1.10, SD: 1.40 ms) versus BF-unaligned (mean: 2.39, SD: 2.51 ms) sites. Based on these findings, it is possible that stimulation of A1 activates the monosynaptic and tonotopic projections to the CNIC that then activate neighboring BF regions through intrinsic connections, leading to the longer latencies for the BF-unaligned versus the BF-aligned sites. Other possible polysynaptic pathways from A1 to the CNIC are presented in the Section “Discussion.”

### CORTICALLY DRIVEN RESPONSES ARE LOCALIZED TO CAUDOMEDIAL CNIC

**FIGURE 4:**
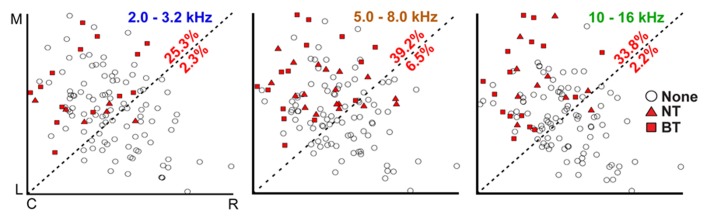
**Corticocollicular activation is localized in the caudomedial portion of the CNIC**. Computer models of isofrequency laminae were created from imaged brain slices and normalized in distance based on the most proximal CNIC site locations in each direction (C, caudal; R, rostral; M, medial; L, lateral) across experiments for low (2.0–3.2 kHz), middle (5.0–8.0 kHz), and high (10.0–16.0 kHz) frequencies. Section “Materials and Methods” for further details and justification for creating these pooled laminae. Percentages correspond to the number of locations in the CNIC showing excitatory responses in the corresponding portion of each lamina without differentiating between NT and BT types (i.e., number of points with NT or BT symbol divided by total number of points in that portion of the lamina). Placements labeled as “None” (open circles) do not necessarily mean that these CNIC locations are never affected by cortical stimulation; instead, they indicate that there was no activation in response to stimulation of the specific A1 locations used for that particular experiment.

To determine whether the NT and BT excitation patterns are evenly distributed across the isofrequency laminae of the CNIC, three-dimensional computer reconstructions of the midbrain based on brain slices were created and normalized across experiments for localization of the CNIC electrode sites. Site locations were superimposed onto a single lamina for low (2.0–3.2 kHz), middle (5.0–8.0 kHz), and high (10.0–16.0 kHz) frequencies. The three isofrequency laminae shown in **Figure 4** have a somewhat elliptical shape as can be visualized by the borders created by the points. Previous studies have shown that the laminae are not square-like but exhibit more complex shapes across layers (Malmierca et al., 1995). A line was drawn from the bottom-left corner to the top-right corner (approximately perpendicular to and in the center along the major axis of these elliptical laminae) to split each lamina into two regions for further analysis as shown in **Figure 4**.

Sites showing excitation (NT or BT patterns) were nearly exclusively located in the caudomedial portion of the CNIC for each of the three isofrequency laminae. In other words, the filled symbols were mainly located within the top-left portion of each box in **Figure 4**. We did not observe any obvious differences in the location of NT (triangles) or BT (squares) activation across each of the laminae, and thus combined those data together for further analysis. From all sites superimposed onto a lamina, we calculated the percentage of those sites that showed excitation (either NT of BT types) in the caudomedial and rostrolateral portions of each lamina. We found that 25.3–39.2% of sites in the caudomedial portion of the CNIC laminae exhibited excitation, while only 2.2–6.5% of sites in the rostrolateral portion were activated.

**FIGURE 5:**
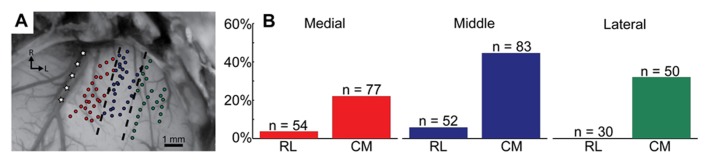
**Different isofrequency locations in A1 show the same caudomedial (CM) to rostrolateral (RL) trend in the CNIC as shown in Figure 4**. Three A1 regions were created by drawing evenly spaced lines parallel to the middle cerebral artery, which is approximately parallel to the tonotopic gradient of A1 (**A**: modified version of **Figure 1**). These three groups (medial in red; middle in blue; lateral in green) correspond to different locations along the isofrequency dimension of A1. The number of BF-matched A1-CNIC shank pairs located within the RL or CM portion of the CNIC are labeled as *n*, and percentages correspond to the number of these pairs exhibiting excitatory activity divided by *n* (**B**). Cortically driven excitation patterns derived from stimulation of all three A1 regions terminate predominantly within the CM portion of CNIC.

The caudomedial activation pattern was also consistent regardless of the stimulated site location across the isofrequency dimension of A1. **Figure 5A** shows how we divided A1 into three regions corresponding to different locations along the isofrequency dimension of A1.We then calculated the percentages of sites that elicited excitation in the caudomedial versus the rostrolateral portion along the three laminae assessed in our study. Regardless of the A1 region, there was always a higher percentage of sites causing excitation in the caudomedial (22.1–44.6%) versus the rostrolateral (0.0–5.8%) portion of the CNIC (**Figure 5B**). These findings suggest the existence of two subregions along the isofrequency dimension of the CNIC that may process sound information through the lemniscal pathway in different ways. In particular, the caudomedial portion compared to the rostrolateral portion of the CNIC may serve a more modulatory role through descending activation from the auditory cortex, involving neurons located throughout A1.

## DISCUSSION

Our results indicate that focal cortical stimulation can induce excitatory responses in the CNIC, in accord with previous studies indicating an excitatory corticocollicular pathway (Feliciano and Potashner, 1995; Zhang and Suga, 1997; Torterolo et al., 1998; Yan and Suga, 1999). These responses can be elicited via stimulation throughout A1, in agreement with studies demonstrating that corticocollicular projections originate across A1 (Bajo and Moore, 2005; Coomes et al., 2005; Schofield, 2009). Also, the descending excitatory pathway, like the ascending lemniscal auditory system (Lorente De Nó, 1981; Malmierca, 2003), is arranged tonotopically and can influence neighboring frequency regions. This organization may provide a potential mechanism for enabling the subcortical frequency plasticity that has been extensively shown in previous studies (Suga, 2008; Xiong et al., 2009; Bajo and King, 2013). Additionally, the responses were found nearly exclusively in the caudomedial CNIC, providing an interesting juxtaposition with the ascending lemniscal auditory system further described below.

### TECHNICAL LIMITATIONS

The use of electrophysiology and invasive brain stimulation has several inherent limitations that need to be discussed for interpreting our results. First, our electrode arrays only allowed us to stimulate and record from a few discrete locations in A1 and the CNIC at any given time. Therefore, several of the values and percentages described in the results could be underestimations of true physiological values. For example, when we stated that 57 out of 80 A1 locations resulted in CNIC excitation, it is possible that a larger proportion of A1 sites would have caused excitation in the CNIC had we been able to more fully map the CNIC for each cortical location. We were only able to record from a few locations (4–5 per animal on average) along a lamina within the CNIC for each cortical location. Similarly, the percentages shown in **Figures 4 and 5** could have been higher if we had been able to sample from a larger number of A1 and CNIC locations.

Second, electrical stimulation can cause complex functional effects by activating a combination of cell bodies and passing fibers (Ranck, 1975; McIntyre and Grill, 2000; McIntyre et al., 2004), especially in highly interconnected regions such as the cortex. We attempted to mitigate these effects by analyzing stimulation levels at or close to activation threshold to limit current spreading across A1. It was typical for activation of CNIC sites to be induced by stimulation of multiple sites along a cortical shank. At our highest stimulation level of 32 μA and using a similar stimulation waveform, current spreading from a stimulated site within brain tissue has shown to activate neurons at an average distance of approximately 100–150 μm (Ranck, 1975; McIntyre and Grill, 2000). Since our sites along a cortical shank were spaced at 200 μm, current spreading may have caused different cortical sites along a shank to activate overlapping neural populations. Also, layer V pyramidal cells, which are the neurons providing the majority of descending projections to the midbrain (Schofield, 2009), have extensive connections along a cortical column that were likely activated by our stimulation (Winer and Prieto, 2001). Therefore, our analysis focused on the cortical site inducing the lowest activation threshold in the CNIC and avoided making comparisons of activation patterns for stimulation of multiple sites along a single cortical shank. The four cortical shanks on each probe, on the other hand, were spaced 500 μm apart and stimulation of sites on different shanks were expected to activate distinct neural populations.

Third, our electrophysiological setup does not allow us to make claims regarding whether the cortically driven excitation in the CNIC was the result of direct or indirect pathways. However, we attempt to describe the potential neural pathways below and, based on our results and previous studies, we postulate the likely sources of this cortically driven excitation in the CNIC.

### TONOTOPIC ACTIVATION OF CNIC NEURONS

Our results indicate that corticocollicular excitatory pathways are arranged in a tonotopic manner. This agrees with several anatomical studies which, when compared with previously published frequency maps within the CNIC, have provided evidence for direct corticofugal projections from A1 to the CNIC that are topographically arranged (Andersen et al., 1980; Feliciano and Potashner, 1995; Saldana et al., 1996; Bajo and Moore, 2005; Coomes et al., 2005; Bajo et al., 2007). However, these studies using anatomical tracers and histological stains inherently could not prove tonotopicity. A neurophysiological study using antidromic stimulation in a similar guinea pig setup functionally confirmed that direct projections from A1 to CNIC are organized in a precise tonotopic pattern (Lim and Anderson, 2007a). Considering the consistency in latency values for our BF-aligned responses with the values published from this antidromic study, it is likely that the tonotopic activation pattern elicited in the CNIC using A1 stimulation is caused by or involves the direct monosynaptic and excitatory corticocollicular projections from A1 to CNIC.

### ACTIVATION OF NEIGHBORING FREQUENCY REGIONS

In the present study, the BF-aligned projections for the NT and BT pathways and their first-spike latencies are consistent with the point-to-point tonotopic corticocollicular projections identified in the previous antidromic study (Lim and Anderson, 2007a). The BF-unaligned sites in the BT pattern, on the other hand, differ from the antidromic data and likely arise from polysynaptic pathways. It is unlikely that the BT pattern was solely the result of current spreading across A1. This expectation is supported by two interesting observations. If the BT excitation pattern was due to current spreading from the stimulated site that then activated neighboring frequency regions in A1 and thus different frequency regions in CNIC via corticofugal pathways, then BF-aligned and BF-unaligned sites should have been activated nearly simultaneously. Instead, the BF-aligned sites had shorter latencies than the BF-unaligned sites. Furthermore, if the BT effect was due to spread of current in A1, then broad activation patterns should also have been observed for the NT pattern for similar current levels.

The BF-unaligned BT projections could, however, arise from several different polysynaptic pathways. (1) Since corticocollicular anatomical projections terminate densely in the DNIC and ENIC**, **one possibility is that cortical stimulation induces excitation in the DNIC and/or ENIC that then projects to the BF-unaligned sites in CNIC. Though the DNIC and ENIC have poor tonotopy (Aitkin et al., 1975; Syka et al., 2000), it is possible that some underlying topographic descending organization exists that could enable frequency-specific activation of neighboring frequency laminae in the CNIC (Saldana et al., 1996). A study in bats showed that cortical activation could modulate activity within the CNIC via the ENIC, though this study did not investigate the tonotopic effects (Jen et al., 2001). (2) Another possibility is that A1 stimulation may activate other nuclei along the auditory pathway that then project to the CNIC. For instance, it is known that electrical stimulation of the auditory cortex can alter coding properties across different frequency regions in the ipsilateral cochlear nucleus (Luo et al., 2008; Liu et al., 2010) and that cortical projections synapse on neurons in the cochlear nucleus that then project to the IC (Schofield and Coomes, 2005). Furthermore, another study showed that two pathways exist from the cochlear nucleus to the IC, a narrow one and a wide one (Malmierca et al., 2005), which may be analogous to the NT and BT patterns described in this study. (3) It is also possible that A1 stimulation activates local cortical interconnections between different frequency regions that then project to the CNIC (Winer and Prieto, 2001) that could contribute to the BT pattern. (4) Based on our data, we suggest that intrinsic projections within the CNIC connecting different isofrequency laminae, as previously described by Malmierca et al. (1995), could explain our BT results. This organization would allow A1 stimulation to activate BF-aligned CNIC neurons that could then activate or modulate neurons within neighboring and even distant frequency regions. The longer latencies observed for the BF-unaligned versus the BF-aligned sites in the BT pattern and the broad but systematic tonotopic pattern observed for the BT pathway are consistent with this proposed descending organization. It is important to note that although the NT pattern consisted of only a single activated BF-aligned site in CNIC, there could also be local projections to neighboring frequency regions that are inhibitory, and thus prevents activation across the tonotopic gradient of the CNIC (Oliver et al., 1994).

### ASCENDING AND DESCENDING LEMNISCAL PATHWAYS

In a previous study in the guinea pig, electrical stimulation of the rostroventral portion (or equivalently the rostrolateral portion) along a CNIC lamina achieved lower thresholds, smaller discriminable level steps, larger evoked potentials, and shorter first-spike latencies in A1 than stimulation of the caudodorsal (or caudomedial) portion (Lim and Anderson, 2007b). Based on those results, the authors suggested that there might exist at least two functional subregions along a given isofrequency lamina of the CNIC that projects in different ways up to the auditory cortex. Interestingly, there are several anatomical and functional studies across species that are consistent with this proposed sub-projection lemniscal pathway. In gerbil, it was shown that brainstem nuclei project in different ways to the caudomedial versus rostrolateral CNIC (Cant and Benson, 2006). In particular, the lateral lemniscus and cochlear nucleus project throughout the CNIC whereas the superior olivary nuclei project predominantly to the rostrolateral region of the CNIC. The rostrolateral CNIC in gerbil was also shown to project predominantly to the rostral portion of the ventral division of the medial geniculate body (MGBv; approximately along the isofrequency dimension) whereas the caudomedial CNIC projects predominantly to the caudal portion of the MGBv (Cant and Benson, 2007). Both in cat and rat, it was shown that the rostral MGBv projects throughout auditory cortex, including A1, but caudal MGBv projects predominantly to regions outside of A1 [e.g., ventral auditory field in rat or posterior auditory field in cat appear to receive more projections from the caudal than the rostral MGBv (Morel and Imig, 1987; Rodrigues-Dagaeff et al., 1989; Storace et al., 2010)]. There is also a functional study showing that in the thalamus of cats in response to acoustic stimulation, neurons in the rostral portion of the MGBv (approximately along the isofrequency dimension) have more precise tonotopy and sharper tuning, are more time-locked, and have shorter latencies than the caudal portion (Rodrigues-Dagaeff et al., 1989). Therefore, based on these results across species, there appears to be two segregated pathways that exist along the ascending lemniscal pathway from the CNIC up to the auditory cortex.

In the current study, we observed that stimulation of A1 resulted in activation predominantly in the caudomedial portion along the isofrequency laminae of the CNIC. Based on the ascending lemniscal organization described above, it is possible that the caudomedial pathway may serve a more modulatory role in the processing of ascending acoustic information while the rostrolateral pathway is involved with robust transmission of acoustic information to A1. It is important to note that different reconstruction techniques were performed across the studies described above, and thus further studies are needed to confirm if the caudomedial versus rostrolateral CNIC regions identified in this study are the same regions identified across those other studies and species (and consistent with the caudal versus rostral pathways through MGBv). Also, different frequency regions were investigated across studies [e.g., 2–16 kHz in this study versus 9–23 kHz in (Lim and Anderson, 2007b)]. However, the consistency in results observed across species in terms of this proposed sub-projection lemniscal organization raises the possibility that it may be a general feature of the mammalian brain. It is also important to note that previous studies in multiple species have shown a differential pattern of excitatory and inhibitory activation in various locations within the CNIC in response to cortical stimulation (Mitani et al., 1983; Syka and Popelar, 1984; Bledsoe et al., 2003). However, these studies did not reconstruct or identify their stimulation and recording sites along the frequency and isofrequency dimensions of the CNIC and A1, and thus further studies across species are still needed to confirm that the caudomedial activation pattern in the CNIC identified in our study is a general feature of the mammalian brain. In addition, the cortically induced suppressive effects across the frequency and isofrequency dimensions of the CNIC also need to be investigated.

### IMPLICATIONS FOR FREQUENCY PLASTICITY

Our findings provide functional evidence for a precise tonotopic organization within the lemniscal corticofugal system that could enable the fine frequency plasticity shown in the CNIC in previous neurophysiological studies (Xiong et al., 2009). Both the NT and BT response patterns show excitation of BF-aligned neurons, consistent with direct corticocollicular projections being glutamatergic (Feliciano and Potashner, 1995) and tonotopic (Saldana et al., 1996; Bajo and Moore, 2005; Lim and Anderson, 2007a). However, the BT pattern also allows A1 to interact with neurons in different frequency regions of the CNIC. In this way, activation of A1 neurons tuned to a specific frequency could cause BF-unaligned CNIC neurons to become more sensitive to that frequency. Other inhibitory pathways into and within the CNIC (Jen et al., 2001; Kelly and Caspary, 2005; Pollak et al., 2011) would likely be involved in altering the tuning selectivity of BF-unaligned CNIC neurons in response to A1 activation. As proposed by several studies (Schofield, 2010; Hormigo et al., 2012; Hurley and Sullivan, 2012), cholinergic, serotonergic, or noradrenergic input from the pontomesencephalic tegmentum, raphe nuclei, or locus coeruleus, respectively, provide neuromodulatory reinforcement directly into the CNIC or indirectly through non-lemniscal midbrain pathways. These different pathways could in turn sustain the spectral changes induced by lemniscal corticofugal activation. Together, these findings provide an initial functional framework for further investigating how modulation and plasticity of different sound features can occur within the central auditory system through spatially organized interactions among the ascending, descending, and neuromodulatory networks (Winer, 2006; Suga, 2008; Xiong et al., 2009).

## Conflict of Interest Statement

The authors declare that the research was conducted in the absence of any commercial or financial relationships that could be construed as a potential conflict of interest.
